# Lymphocyte Function at Baseline Could Be a New Predictor of Tumor Burden following Six Cycles of Radium-223 Therapy in Patients with Metastasized, Castration-Resistant Prostate Cancer

**DOI:** 10.3390/cancers16050886

**Published:** 2024-02-22

**Authors:** Vahé Barsegian, Daniel Möckel, Sebastian Buehler, Stefan P. Müller, Michael C. Kreissl, Patrick Ostheim, Peter A. Horn, Monika Lindemann

**Affiliations:** 1 Institute of Nuclear Medicine, Helios Kliniken, 19055 Schwerin, Germany; vahe.barsegian@helios-gesundheit.de (V.B.); daniel.moeckel@helios-gesundheit.de (D.M.); sebastian.buehler@helios-gesundheit.de (S.B.); 2 Department of Nuclear Medicine, University Hospital, 45147 Essen, Germany; 3 Division of Nuclear Medicine, Department of Radiology and Nuclear Medicine, Otto von Guericke University, 39106 Magdeburg, Germany; michael.kreissl@med.ovgu.de; 4 Bundeswehr Institute of Radiobiology, 80937 Munich, Germany; patrickostheim@bundeswehr.org; 5 Institute for Transfusion Medicine, University Hospital, 19055 Essen, Germany; peter.horn@uk-essen.de

**Keywords:** radium-223 therapy, antimicrobial immune response, lymphocyte proliferation, ELISpot, interferon-γ, interleukin-10, prediction of tumor burden

## Abstract

**Simple Summary:**

Patients with metastasized, castration-resistant prostate cancer can be treated locally with radioactive radium-223, which usually comprises six cycles. We wanted to know whether this treatment affects immune function. We performed cell culture experiments with white blood cells from the patients and added components of microorganisms. We tested the ability of the cells to proliferate and produce two cytokines (interferon-gamma and interleukin-10) that are important for the balance of the immune system. Our data in 21 patients indicate that the immune cells show impairment in their defense against microorganisms after treatment. As determined by bone scintigraphy, a reduction in tumor burden was observed in 67% of patients. Interestingly, even before treatment, the number of cells producing interleukin-10, an anti-inflammatory cytokine, was an indicator of tumor burden at the end of treatment. Thus, a blood test can help assess whether the treatment is likely to be successful.

**Abstract:**

Previous data indicate that one cycle of treatment with radium-223 (^223^Ra) did not significantly impair lymphocyte function in patients with metastasized, castration-resistant prostate cancer. The aim of the current study was to assess in 21 patients whether six cycles of this therapy had an effect on lymphocyte proliferation and interferon-γ and interleukin (IL)-10 ELISpot results. Lymphocyte proliferation after stimulation with microbial antigens and the production of interferon-γ continuously decreased after six cycles of radionuclide therapy, reaching statistical significance (*p* < 0.05) at months 1, 2, 4, and/or 6 after therapy. One month after the last cycle of therapy, 67% of patients showed a decrease in tumor burden. The tumor burden correlated negatively with IL-10 secretion at baseline, e.g., after stimulation with tetanus antigen (*p* < 0.0001, *r* = −0.82). As determined by receiver operating characteristic (ROC) curve analysis, tetanus-specific IL-10 spots at baseline had the highest predictive value (*p* = 0.005) for tumor burden at month 6, with an area under the curve (AUC) of 0.90 (sensitivity 100%, specificity 78%). In conclusion, we observed an additive effect of treatment with ^223^Ra on immune function and found that IL-10 secretion at baseline predicted tumor burden at month 6 after treatment.

## 1. Introduction

Prostate cancer is the most common cancer in men, and one out of eight males develops invasive prostate cancer during their lifetime [1]. To date, the mortality from prostate cancer is 18.8 out of 100,000 men [1]. Prostate cancer metastasizes most frequently to local lymph nodes and the skeleton, and the presence of bone metastases is an impactful prognostic factor [2].

The administration of the alpha-emitting radioisotope radium-223 chloride (^223^Ra, Xofigo^®^, Bayer Vital GmbH, Leverkusen, Germany) [3] is an innovative treatment option for patients with castration-resistant prostate cancer with skeletal metastases. ^223^Ra is a calcium analogue that is integrated into the bone, and it can emit four alpha particles [4,5]. Previous data showed that ^223^Ra therapy can prolong patient survival and reduce pain [6,7,8]. ^223^Ra penetrates only slightly into the tissue but has a high amount of energy per track length [9]. It is mainly excreted via the small intestine [10] so that it can be used regardless of kidney function.

The side effects of ^223^Ra therapy include vomiting, diarrhea, and myelosuppression leading to leukopenia and thrombocytopenia [9]. However, compared to beta emitters, the toxic effects on hematopoiesis are less frequent with ^223^Ra [9]. It has been published that radiotherapy with beta and gamma isotopes induces numerical and also functional changes in leukocytes [11,12,13,14,15]. In line with only mild effects of ^223^Ra, our group showed that in eleven patients with metastasized, castration-resistant prostate cancer one cycle of treatment with this alpha-emitter did not significantly impair lymphocyte function in vitro [16]. However, in patients with ankylosing spondylitis treatment with ^223^Ra led to a reduction of inflammatory responses (C-reactive protein) [17]. Malamas et al. exposed human prostate carcinoma cells in vitro to sublethal doses of ^223^Ra and found significantly enhanced T cell-mediated lysis of tumor cells by CD8+ cytotoxic T cells [18], indicating a favorable effect of ^223^Ra on antitumor control.

As compared to a single cycle of ^223^Ra therapy, consecutive cycles may have an additive effect on hematopoietic toxicity and may thus lead to detectable effects on in vitro immune function in patients with metastatic prostate cancer. Due to its high sensitivity, the lymphocyte transformation test (LTT) is considered the gold standard for the determination of lymphocyte proliferation after irradiation [11,12,14,15,19,20,21,22] and was also used in our previous study on antimicrobial immune function after one cycle of ^223^Ra therapy [16]. The LTT determines cell proliferation by measuring the incorporation of ^3^H-thymidine during DNA duplication, e.g., after stimulation with mitogens or recall antigens.

While mitogens stimulate the proliferation of both naïve and memory T cells, microbial antigens (recall antigens) only stimulate memory T cells [12]. A normal response to mitogens is an indication of an overall intact reactivity of the lymphocytes, and a normal response to microbial antigens indicates adequate recognition of microorganisms by antigen-presenting cells and T cells. In addition to cell proliferation, the production of cytokines can be determined, using the highly sensitive enzyme-linked immunospot (ELISpot) assay. The ELISpot assay quantifies the effects of irradiation on a single cell level [11,12,14,15,16,23,24]. We have chosen key pro- and anti-inflammatory cytokines and characterized the T helper type 1 response by interferon (IFN)-γ secretion and the T helper type 2 response by interleukin (IL)-10 secretion.

The aim of this study was to determine if six cycles of therapy with ^223^Ra induced an impairment of cellular in vitro immune responses, which has not yet been published. To measure T cell function, we isolated peripheral blood mononuclear cells (PBMC) and stimulated the cells with mitogens and microbial antigens. We then determined T cell proliferation and the production of IFN-γ and IL-10 by ELISpot assay. Twenty-one patients with metastatic prostate cancer were tested sequentially before treatment with ^223^Ra and at months 1, 2, 4, and 6 after treatment. In parallel, blood cell counts, hemoglobin concentration, and tumor burden were determined. Finally, we analyzed, if clinical parameters (blood cell counts and hemoglobin concentration, tumor burden, and age) correlated with cellular in vitro immune function.

## 2. Materials and Methods

### 2.1. Patients

From June 2017 to December 2020, 21 patients with metastatic castration-resistant prostate cancer, with a mean age of 70 years, were consecutively enrolled in this study (Table 1) and received treatment with ^223^Ra (Xofigo^®^). The patients had bone or lymph node metastases, but no metastases elsewhere. Skeletal scintigraphy was performed prior to therapy for treatment planning. The ^223^Ra activity used was 50 kBq/kg body weight. Cellular immunity was analyzed immediately before therapy (month 0) and at months 1, 2, 4, and 6 after the first cycle of therapy. At month 1 the patients had received one cycle of therapy (at month 0), at month 2 two cycles (at month 0 and month 1), at month 4 four cycles (at month 0, 1, 2, and 3), and at month 6 six cycles (at month 0–5). Follow-up had to be skipped during the first months of the COVID-19 pandemic because the lab was involved in diagnostics of SARS-CoV-2 immunity. Thereby, at month 1, cellular in vitro data were missing in one patient, at month 2 in five, at month 4 in six, and at month 6 in seven. However, the exclusion of the incomplete data sets yielded comparable results to those displayed in the figures. In parallel to cellular in vitro assays, leukocyte, thrombocyte, and erythrocyte counts as well as the hemoglobin concentration were measured. Moreover, the patients were asked for infections by a questionnaire at months 1, 3, 4, and 6 after ^223^Ra therapy.

The study was approved by the local ethics committee (09-3991) and it was performed in accordance with the principles of the 1964 Declaration of Helsinki and its later amendments. Written informed consent was obtained from all individual participants included in the study.

### 2.2. Lymphocyte Transformation Test

The proliferation of PBMC was determined after stimulation with the mitogens phytohemagglutinin (PHA), concanavalin A, pokeweed mitogen (PWM), and anti-CD3 monoclonal antibody (OKT3), using standardized assay formats [11,25]. Moreover, responses to the microbial antigens tuberculin (75 µg/mL), *Candida albicans* (130 µg/mL), and Herpes simplex virus type 1 (1:16 dilution of an antigen for complement binding reactions) were measured, as described [26]. Autologous (unstimulated, negative) controls were grown in parallel. In brief, after density gradient centrifugation 50,000 PBMC per 200 µL were incubated in separate cultures for 3 days with four mitogens or for 5 days with three microbial antigens, respectively. For the last 16 h, 37 kBq ^3^H thymidine was added to the cell cultures, to determine cell proliferation. Cells were then lysed, the cell debris was transferred to filter pads, and liquid scintillation counting was performed to quantify the incorporated radioactivity.

### 2.3. ELISpot Assay

IFN-γ and IL-10 ELISpot assays were performed as described previously in detail [11]. Stimulation with four mitogens (same as for LTT) and with three microbial antigens [tuberculin (75 µg/mL), tetanus toxoid (25 µg/mL), and *Candida albicans* (130 µg/mL)] was performed for 2 days. Negative controls (unstimulated cells) were cultured in parallel. To assess the response to mitogens and antigens, 200,000 and 400,000 PBMC per 200 µL cell culture were grown, respectively. Cytokine spots were measured by an ELISpot plate reader (AID Fluorospot, Autoimmun Diagnostika GmbH, Strassberg, Germany).

### 2.4. Assessment of Tumor Burden

The automated Bone Scan Index (BSI) technology from EXINI [27] was used to quantify the tumor burden, prior to therapy and at months 3 and 6 after starting the therapy. It is a quantitative, reproducible tool for estimating the burden of bone metastases in patients with advanced prostate cancer [28]. The BSI is defined by the percentage of tumors in the total skeletal mass. It is calculated by adding the proportion of each bone, expressed as a percentage of the total skeleton [29]. For example, a BSI value of 1 means that a tumor has affected 1% of the entire skeleton.

### 2.5. Statistical Analysis

Statistical analyses were performed using IBM SPSS Statistics version 25 (Armonk, New York, NY, USA) or GraphPad Prism 8.4.2.679 (San Diego, CA, USA). LTT results are expressed as counts per minute (CPM), i.e., as incorporation of ^3^H thymidine, and ELISpot results as cytokine spots per cell culture. Unless otherwise stated, the mean and standard error of the mean (SEM) are given. The responses in months 0, 1, 2, 4, and 6 were compared using one-way analysis of variance (ANOVA), applying a mixed-effects model, and *p* values were corrected for multiple comparisons, using Dunnett’s post-hoc test. Spearman analysis was performed to assess the correlation between clinical parameters (blood cell counts, hemoglobin concentration, BSI, age) and immune function. The predictive value of the ELISpot results was analyzed by receiver operating characteristic (ROC) curve analysis. The endpoint was the occurrence of high tumor burden (>median) at month 6 after starting treatment with ^223^Ra. Analyses were performed two-sided and considered significant at *p* < 0.05.

## 3. Results

### 3.1. Immune Response after Radium-223 Therapy

**Figure 1:**
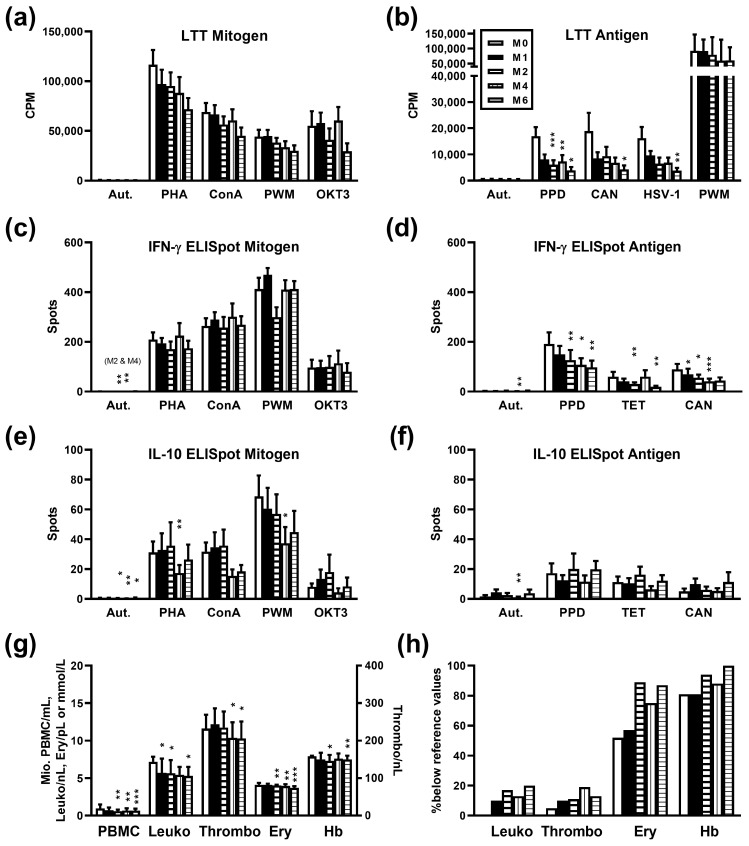
Cellular in vitro immune responses, blood cell counts, and hemoglobin concentrations in 21 patients with metastasized prostate cancer receiving radium-223 therapy. Data represent the mean and standard error of the mean (SEM), either pre-therapy (month 0, white bars) or at months (M) 1 to 6 post-therapy. Panels (**a**,**b**) indicate proliferative responses (lymphocyte transformation test, LTT), panels (**c**–**f**) ELISpot results (spots per culture), and panel (**g**,**h**) blood cell counts and hemoglobin (cell number and concentration (**g**) and percentage of patients with values below local reference values (**h**), respectively). In none of the patients, leukocyte numbers were below the normal range prior to therapy. Data at various time points were analyzed by One-way ANOVA, applying a mixed-effects model, and *p* values were corrected for multiple comparisons, using Dunnett’s post-hoc test (* *p* < 0.05, ** *p* < 0.01, *** *p* < 0.001). CPM, counts per minute; Aut., autologous (negative control); PHA, phythohemagglutinin; ConA, concanavalin A; PWM, pokeweed mitogen (used also as a positive control for the LTT antigen); OKT3, anti-CD3 monoclonal antibody; PPD, purified protein derivate (tuberculin); CAN, *Candida albicans*; HSV-1, Herpes simplex virus type 1; TET, tetanus toxoid; IFN, interferon; IL, interleukin; PBMC, peripheral blood mononuclear cells; Leuko, leukocytes per nL heparinized blood; Thrombo, thrombocytes per nL; Ery, erythrocytes per pL (= million per µL); Hb, hemoglobin concentration (mmol/L).

Twenty-one patients with metastasized, castration-resistant prostate cancer were analyzed prior to therapy with ^223^Ra and at months 1, 2, 4, and 6 thereafter by LTT and ELISpot, to quantify lymphocyte proliferation and cytokine secretion, respectively. In contrast to mitogen-induced lymphocyte proliferation which was only non-significantly reduced after therapy (Figure 1a), proliferation after stimulation with three microbial antigens declined significantly at month 6 (*p* < 0.05) (Figure 1b). In detail, proliferative responses towards tuberculin (purified protein derivate, PPD) decreased from a mean value of 16,990 to 3893 CPM, towards *Candida albicans* from 18,935 to 4228 CPM and HSV type 1 from 16,215 to 3790 CPM. In addition, responses towards tuberculin were significantly (*p* < 0.01) reduced at months 2 and 4 (6125 and 7317 CPM, respectively). The secretion of the pro-inflammatory cytokine IFN-γ did not change significantly after stimulation with mitogens (Figure 1c). However, similar to data on lymphocyte proliferation, IFN-γ secretion declined significantly after stimulation with microbial antigens (Figure 1d). At month 1, the decline already reached statistical significance for responses to *Candida albicans* (*p* < 0.05), at month 2 for tuberculin and tetanus toxoid (*p* < 0.01). The secretion of the anti-inflammatory cytokine IL-10 showed a significant decrease (*p* < 0.05) at month 4 after stimulation with the mitogens PHA and PWM (Figure 1e). However, the pattern was less clear as compared to data on IFN-γ. IL-10 secretion remained overall constant after stimulation with microbial antigens (Figure 1f).

Thus, our main finding was that after stimulation with microbial antigens lymphocyte proliferation and the production of IFN-γ decreased after six cycles of ^223^Ra therapy. At month 6, for tuberculin only 23% (3893 CPM/16,990 CPM), for *Candida albicans* 22%, and for HSV-type 1 23% of baseline proliferation was reached. IFN-γ production after stimulation with microbial antigens decreased to 51% (tuberculin), 32% (tetanus toxoid), and 49% (*Candida albicans*) at month 6 after therapy. Nevertheless, in a structured questionnaire, none of the patients reported infections after ^223^Ra therapy.

### 3.2. Influence of Radium-223 Therapy on Blood Cell Counts and Hemoglobin Concentration

As compared to baseline, the number of PBMC isolated per mL blood, leukocyte, thrombocyte, erythrocyte count, and hemoglobin concentration were significantly (*p* < 0.05) reduced at month 6 after therapy (Figure 1g). In detail, PBMC declined from 0.97 ± 0.11 to 0.67 ± 0.07 million per mL heparinized blood, leukocytes from 7.2 ± 0.7 to 5.4 ± 0.5/nL (reference values 3.8–9.6), thrombocytes from 257 ± 21 to 208 ± 14/nL (reference values 150–430), erythrocytes from 4.1 ± 0.1 to 3.6 ± 0.1 million/µL (reference values 4.2–5.4) and hemoglobin concentration from 7.6 ± 0.2 to 7.1 ± 0.3 mmol/L (reference values 8.6–12.0) (data represent mean and SEM). A significant (*p* < 0.05) reduction of leukocyte count was already detectable at month 1, of PBMC, erythrocyte count, and hemoglobin concentration at month 2, and of thrombocyte count at month 4. At baseline, in none of the patients, leukocytes were below the reference values, in 5% thrombocytes, in 52% erythrocytes, and in 81% hemoglobin concentrations. At month 6 after therapy, the respective numbers increased to 20%, 13%, 87%, and 100% (Figure 1h).

### 3.3. Influence of Radium-223 Therapy on Tumor Burden

The tumor burden, i.e., the percentage of total skeletal mass taken up by the tumors, prior to therapy and at month 3 was similar (2.5 ± 0.5 and 2.5 ± 0.6), at month 6 it declined non-significantly to 1.9 ± 0.4 (mean ± SEM). Comparing data prior to therapy and at month 3, 42% of patients showed a decline. Comparing data prior to therapy and at month 6, 67% of patients showed a decline. At month 6, the median tumor burden was 89% of baseline values (range 3–544%). Individual courses of BSI values are shown in Table 1.

### 3.4. Correlation Analysis of Clinical Parameters and Cellular In Vitro Immune Responses

**Figure 2:**
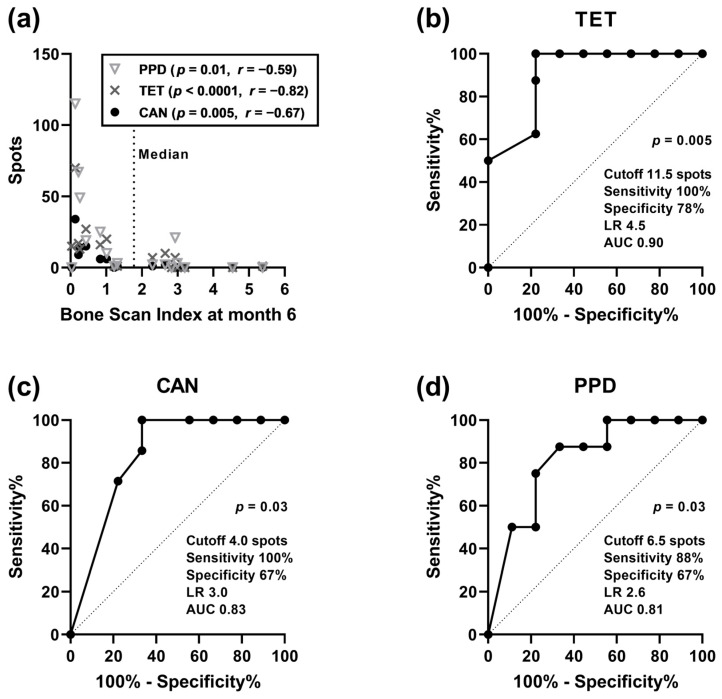
Correlation between tumor burden at month 6 after therapy with radium-223 and IL-10 ELISpot results at baseline in patients with metastasized prostate cancer. The tumor burden was quantified by the Bone Scan Index. Data on IL-10 ELISpot results were considered after stimulation with the antigens tuberculin (purified protein derivate, PPD, *n* = 17 data pairs), tetanus toxoid (TET, *n* = 17) and *Candida albicans* (CAN, *n* = 16). Panel (**a**) indicates the results of a Spearman correlation analysis and panels (**b**–**d**) a receiver operating characteristic (ROC) curve analysis. LR, likelihood ratio; AUC, Area under curve.

Spearman analysis indicated that there was a significant, negative correlation (*p* < 0.05) between BSI values (at baseline, month 3 and 6 after therapy) and IL-10 production in the autologous controls and after stimulation with tuberculin, tetanus toxoid, and *Candida albicans* at baseline (Table 2). The highest level of significance was obtained for BSI values at month 6 (tuberculin: *p* = 0.01, tetanus toxoid: *p* < 0.0001, Candida albicans: *p* = 0.005) (Figure 2a). Thus, patients with higher tumor burden despite therapy had the lowest IL-10 production already at baseline. A similar trend for negative correlation was observed for lymphocyte proliferation and IFN-γ production at baseline (autologous control and response to microbial antigens). Thus, in patients with higher tumor burden, the production of anti- and pro-inflammatory cytokines and lymphocyte proliferation were more severely impaired, pointing to a less active immune system.

As BSI values at month 6 after therapy and IL-10 secretion after stimulation with microbial antigens at baseline showed the strongest correlation, we further analyzed these data by ROC curve analysis. We divided the patients according to the tumor burden at month 6 and used the median value as the cutoff (BSI of 1.755). IL-10 spots after stimulation with tetanus toxoid had the highest predictive value (*p* = 0.005) for the occurrence of high tumor burden (>median), with an area under the curve (AUC) of 0.90 (cutoff 11.5 spots: sensitivity 100%, specificity 78%, likelihood ratio (LR) 4.5), followed by responses after stimulation with *Candida albicans* (*p* = 0.03, AUC 0.83, sensitivity 100%, specificity 67%) and tuberculin (*p* = 0.03, AUC 0.81, sensitivity 88%, specificity 67%) (Figure 2b–d). Thus, our data indicate that the secretion of IL-10 was predictive of tumor burden after six cycles of therapy.

In addition, BSI values at baseline and erythrocyte count correlated negatively, which reached statistical significance (*p* < 0.05) at baseline and months 2 and 4 after therapy (*r* = −0.42, *r* = −0.54 and *r* = −0.61, respectively). A similar negative correlation (*p* < 0.05) could be observed for BSI values at baseline and hemoglobin concentrations at various time points (baseline: *r* = −0.57; month 1: *r* = −0.45; month 2: *r* = −0.67, month 4: *r* = −0.62). These results suggest a relationship between higher tumor burden and tumor-associated anemia. Finally, patient age did not correlate with cellular immunity or with tumor burden, neither prior to nor post-therapy.

## 4. Discussion

The current study on patients with metastasized, castration-resistant prostate cancer shows for the first time that consecutive cycles of treatment with ^223^Ra led to a significant reduction of lymphocyte function. In addition, the data on immune function prior to treatment are of interest from a clinical point of view, because an in vitro marker predicting outcome after 6 months of ^223^Ra therapy could still not be established. Our results indicate that patients with higher tumor burden (defined by BSI) already at baseline displayed lower autologous (spontaneous) and antigen-specific IL-10 secretion. According to a ROC curve analysis, IL-10 spots after stimulation with tetanus toxoid were highly predictive of tumor burden (*p* = 0.005, AUC 0.90, sensitivity 100%, and specificity 78%). Thus, if confirmed by independent studies from other centers, an IL-10 ELISpot assay could be used as a new tool to predict the treatment response after ^223^Ra therapy. Nevertheless, we re-analyzed an independent group of eleven patients with metastasized, castration-resistant prostate cancer receiving one cycle of treatment with ^223^Ra and found exactly the same phenomenon, a negative correlation between tumor burden and antigen-specific IL-10 secretion at baseline [16]. A correlation between high tumor burden and low anti-inflammatory response (IL-10 secretion) was unexpected at first glance. Nevertheless, it is in line with previous data by Tanikawa et al. showing that IL-10 ablation promotes tumor development, growth, and metastasis [30]. They studied tumor growth in IL-10-deficient (IL-10−/−) mice and found that endogenous IL-10 inhibits inflammatory cytokine production and hampers the development of Treg cells and myeloid-derived suppressor cells, two key components of the immunosuppressive tumor microenvironment, thereby inhibiting tumor development, growth, and metastasis. It was observed that the increased tumor development in IL-10−/− mice resulted from multiple intertwined mechanisms: increased immunosuppression, enhanced inflammation, and possible reduced effector T cell function and tumor trafficking in tumor-bearing hosts. They discussed that the biological activity of IL-10 could be highly context-dependent.

In addition, a previous study on DOTA(0)-Phe(1)-Tyr(3)-octreotide (DOTATOC) treatment in patients with neuroendocrine tumors showed that the presence of more advanced tumors, with bone metastases, correlated with impaired lymphocyte responses to microbial antigens [12]. Moreover, in the current study age did not correlate significantly with lymphocyte function, most likely due to the rather narrow age range (53–83 years) of the patients.

Extending our previous data on a follow-up period of 28 days (after one cycle of treatment) in 11 patients, which did not show a significant decrease in immune function after therapy [16], the current study indicates an additive effect of six cycles of treatment with ^223^Ra. Moreover, this independent, larger cohort could already show a significant impact of ^223^Ra therapy at month 1 for the IFN-γ ELISpot after stimulation with *Candida albicans*. Most likely, immune cells in the close vicinity of osseous metastasis will be destroyed by ^223^Ra, alpha particles that are integrated into the skeleton. However, due to its very short tissue range of <0.1 mm [31], it must be assumed that only a small proportion of immune cells is reached by the radionuclide. Nevertheless, we could measure a decline in blood cell counts (leukocytes, thrombocytes, erythrocytes), indicating myelosuppression as already described in the current guideline “Radionuclide therapy of bone metastases using radium-223” [32]. According to this guideline, thrombocyte count in patients prior to therapy has to be greater than 100/nL, a limit that was not undercut in any of our patients at any time point, even not at month 6 after therapy. After the sixth cycle of therapy, the leukocytes decreased to 75% of the baseline value, thrombocytes to 81%, and erythrocytes to 88%. Thrombocytopenia occurred in 15% of the patients after the sixth cycle of therapy, decreased erythrocyte count in 87%, and decreased hemoglobin concentrations in 100%. However, already at baseline, hemoglobin concentrations were below the normal range in 81% of the patients. Similar to our data, in a large study on castration-resistant prostate cancer and bone metastases (ALSYMPCA) [6], thrombocytopenia occurred in 69 out of 600 patients (12%) after six cycles of ^223^Ra therapy. Moreover, at baseline, the ALSYMPCA study reported a median hemoglobin concentration of 12.2 g/dl (7.6 mmol/L), which is very close to the current data (median 7.9 mmol/L, mean 7.6 mmol/L, normal range 8.6–12.0). Most likely, anemia was observed at a high frequency because according to our current guideline [32], the administration of ^223^Ra is generally not a curative, but a palliative treatment with the intention of prolonging life and reducing osseous pain/complications, i.e., it is applied at an advanced stage of prostate cancer.

We assume that two different effects occur after ^223^Ra therapy. On the one hand, absolute leukocyte and erythrocyte counts decrease because the cells are destroyed by alpha particles, that deliver a high quantity of energy per track length. However, the functional assays were performed with a defined number of PBMC. Thus, on the other hand, cells show an impairment of function, especially after several cycles of treatment. Most likely, this impairment occurs in the more distant vicinity of ^223^Ra, where radiation does not lead to cell death but to functional alterations of cells.

## 5. Conclusions

Our findings demonstrate for the first time that six cycles of therapy with the alpha-emitter ^223^Ra in patients with metastatic castration-resistant prostate cancer impaired antimicrobial T cell responses in vitro. Nevertheless, as compared to patients with hepatic malignancies receiving selective internal radiotherapy, immunosuppression after ^223^Ra therapy was moderate [14]. This is comforting from a clinical point of view, as is the fact that the patients did not report infectious complications after therapy. Moreover, in the current cohort, IL-10 secretion at baseline was predictive of tumor burden after six cycles of ^223^Ra therapy, indicating that performing an IL-10 ELISpot prior to treatment may help to decide if this therapy should really be applied, especially in patients with numerous comorbidities.

## Figures and Tables

**Table 1:** Characteristics of 21 patients with metastasized prostate cancer receiving radium-223 therapy.

ID	Age	BSI 0	BSI M3	BSI M6	% Change M6 vs. 0
1	78	3.85	2.55	2.83	74
2	76	2.64	3.27	2.30	87
3	83	4.62	3.51	4.54	98
4	57	0.87	0.78	0.83	95
5	66	0.39	0.73	1.22	313
6	66	3.32	2.75	2.65	80
7	66	1.12	0.37	0.03	3
8	77	5.54	4.53	2.93	53
9	75	0.35	0.26	0.22	63
10	70	2.56	2.75	3.20	125
11	60	7.12	N.A.	N.A.	N.A.
12	77	0.10	0.34	0.26	260
13	76	0.55	2.79	2.99	544
14	71	1.27	1.35	0.43	34
15	53	0.30	0.15	0.13	43
16	56	3.89	4.45	5.38	138
17	78	2.63	N.A.	N.A.	N.A.
18	65	6.68	10.98	1.31	20
19	68	1.01	1.07	1.01	100
20	80	1.59	2.04	STOP	N.A.
21	75	2.44	2.87	2.2	90

BSI, Bone Scan Index at baseline (0), month 3 (M3), and month 6 (M6) after therapy; N.A., not available; STOP, therapy, and follow-up were stopped due to rapid deterioration of the patient’s clinical condition.

**Table 2:** Spearman analysis between tumor burden (Bone Scan Index) and IL-10 ELISpot results in patients with metastasized prostate cancer receiving radium-223 therapy.

IL-10 ELISpot at Baseline	BSI 0	(*n* = 20)	BSI M3	(*n* = 18)	BSI M6	(*n* = 16–17)
	*p*	*r*	*p*	*r*	*p*	*r*
Autologous	0.06	−0.43	0.02	−0.54	0.01	−0.60
Tuberculin	0.01	−0.55	0.07	−0.43	0.01	−0.59
Tetanus toxoid	0.03	−0.49	0.008	−0.60	<0.0001	−0.82
*Candida albicans*	0.02	−0.54	0.01	−0.59	0.005	−0.67

BSI, Bone Scan Index at baseline (0), month 3 (M3), and month 6 (M6) after therapy.

## Data Availability

The data presented in this study are available on request from the corresponding author. The data are not publicly available due to privacy restrictions.
